# Optimizing the diagnosis and management of dementia within primary care: a systematic review of systematic reviews

**DOI:** 10.1186/s12875-021-01461-5

**Published:** 2021-08-11

**Authors:** Brooklynn Fernandes, Zahra Goodarzi, Jayna Holroyd-Leduc

**Affiliations:** 1 Faculty of Science, University of Calgary, Calgary, Canada; 2 Departments of Medicine and Community Health Sciences, Cumming School of Medicine, Foothills Medical Centre, University of Calgary, North Tower (Rm 930), 1403 29 St NW, Calgary, AB T2N 2T9 Canada

**Keywords:** Dementia, Primary care, Family physician, Systematic review, Diagnosis

## Abstract

**Background:**

To understand how best to approach dementia care within primary care and its challenges, we examined the evidence related to diagnosing and managing dementia within primary care.

**Methods:**

Databases searched include: MEDLINE, Embase, PsycINFO and The Cochrane Database of Systematic Reviews from inception to 11 May 2020. English-language systematic reviews, either quantitative or qualitative, were included if they described interventions involving the diagnosis, treatment and/or management of dementia within primary care/family medicine and outcome data was available. The risk of bias was assessed using AMSTAR 2. The review followed PRISMA guidelines and is registered with Open Science Framework.

**Results:**

Twenty-one articles are included. The Mini-Cog and the MMSE were the most widely studied cognitive screening tools. The Abbreviated Mental Test Score (AMTS) achieved high sensitivity (100 %, 95 % CI: 70-100 %) and specificity (82 %, 95 % CI: 72-90 %) within the shortest amount of time (3.16 to 5 min) within primary care. Five of six studies found that family physicians had an increased likelihood of suspecting dementia after attending an educational seminar. Case management improved behavioural symptoms, while decreasing hospitalization and emergency visits. The primary care educational intervention, Enhancing Alzheimer’s Caregiver Health (Department of Veterans Affairs), was successful at increasing carer ability to manage problem behaviours and improving outcomes for caregivers.

**Conclusions:**

There are clear tools to help identify cognitive impairment in primary care, but strategies for management require further research. The findings from this systematic review will inform family physicians on how to improve dementia diagnosis and management within their primary care practice.

**Supplementary Information:**

The online version contains supplementary material available at 10.1186/s12875-021-01461-5.

## Background

At any given time, 5–8 % of the general population aged 60 and over are living with dementia, and it is expected that 152 million people in the world will have dementia by 2050 [1]. The impact of dementia is far reaching, as it affects not only the person with dementia, but also their family carers, the healthcare system and society as a whole [1]. Dementia is often unrecognized, and there is an underuse of diagnostic assessment tools and a lack of attention to the issues faced by family caregivers [2]. Approximately 65 % of dementia cases are undiagnosed in primary care, which negatively impacts these patients by not implementing advanced care planning and management strategies before the dementia progresses [3]. The U.S Preventative Services Task Force recommends that clinicians assess cognitive functioning when a patient is suspected of cognitive impairment based on the physician’s observation or caregiver concerns [3]. Canadian consensus guidelines similarly do not recommend asymptomatic screening, but instead suggest use of validated screening tools if there is clinical concern for a cognitive disorder [4]. Common neuropsychological screening tools administered by family physicians (FPs) include the Mini-Mental State Examination (MMSE) and Clock Drawing Test (CDT) [3]. However, it is not clear that these are the best screening tools for use in primary care.

Time constraints are often an issue for family doctors as it relates to diagnosing and managing dementia. The time allocated for a typical office visit makes it challenging to perform a cognitive assessment [5]. FPs often feel uncertainty regarding the management of dementia after a diagnosis has been made [5]. This highlights the current need to better optimize dementia care within primary care. The objective of this systematic review of systematic reviews was to determine the most effective evidence-based strategies to diagnose and manage dementia within primary care. Specifically, we seek to understand what practices FPs can undertake to ensure accurate and timely testing and management.

## Methods

This systematic review was conducted in accordance to *PRISMA* (Preferred Reporting Items for Systematic Reviews and Meta-analyses) guidelines [6], and the protocol is registered in Open Science Framework [DOI 10.17605/OSF.IO/E4AW5]. All data generated or analysed during this study are included in this published article in Additional file 1: Appendixes 1 and 2. A systematic review of systematic reviews was determined to be the past method to further summarize and tailor the current body of literature on this topic into a format that would address the existing evidence to practice gap.

### Data Sources

The systematic literature search was developed in consultation with a health sciences librarian, with the final search being completed 11 May 2020. The following databases using the Ovid platform were searched without a restriction to publication date: MEDLINE, EMBASE, PsycINFO and *The Cochrane Database of Systematic Reviews*. We searched the following clusters of search terms: *Family Practice* and *Dementia*. In each category, we used controlled vocabulary such as Medical Subject Headings (MeSH) as well as keywords. Within each cluster, terms were combined with OR, and between the clusters with AND. We then used CADTH search terms for the systematic review study designs [7] (Additional file 1: Appendix 1). The reference list of a previous relevant systematic review of systematic reviews published in 2014 was also searched [8].

### Study Selection

Systematic reviews were considered if they met the following inclusion criteria.

- Population: Primary care or family practice settings seeing persons with dementia.
- Intervention: The detection, diagnosis, treatment and/or management of dementia including models of care, pathways and/or protocols.
- Comparators: Usual care, wait-list control or other interventions within the scope of the review.
- Outcomes: The description of the detection, diagnosis, treatment or management strategies, along with measures of their acceptability, efficacy or effectiveness in the provision of care.
- Study design: Systematic review, either quantitative or qualitative.

Articles were also selected for inclusion if they were English-language articles, included relevant descriptions of the interventions used, and outcome data was available.

Two reviewers (B.F and J.H.-L.) independently screened the titles and abstracts for possible inclusion. If either reviewer thought the citation was relevant or potentially relevant, the full-text article was then retrieved for further evaluation. All full-text articles were assessed independently for inclusion by B.F and J.H.-L. Any conflicts were resolved through discussion. One reviewer (B.F.) independently extracted the following information from the included full-text studies using a standardized data extraction form: authors, year of publication, country where the review was conducted, number of studies included, study designs included, databases searched, time frame of article search, inclusion and exclusion criteria, population (mean age, SD and dementia diagnosis), intervention, comparator, sample size, setting (if the intervention was cognitive screening, the method of administration), time of administration (if intervention was cognitive screening), cognitive outcome(s) measured, results (meta-analysis, Sn, Sp, accuracy), and other (Additional file 1: Appendix 2). One reviewer (B.F) categorized each study based on the primary category of intervention, which was verified by another reviewer (J.H-L).

### Quality Assessment and Analysis

Two reviewers (B.F and J.H.-L.) independently assessed the quality of the included studies using the AMSTAR 2 Systematic Review Quality Appraisal Checklist 2020. Systematic reviews without a clear PICO were excluded. Best practices for quality assessment using AMSTAR 2 are to consider the impact of inadequate ratings for each category rather than generate an overall score. The AMSTAR 2 quality appraisal results for each of the included studies is available in Additional file 1: Appendix 3 [9]. A qualitative descriptive summary of the literature is presented.

## Results

**Fig. 1:**
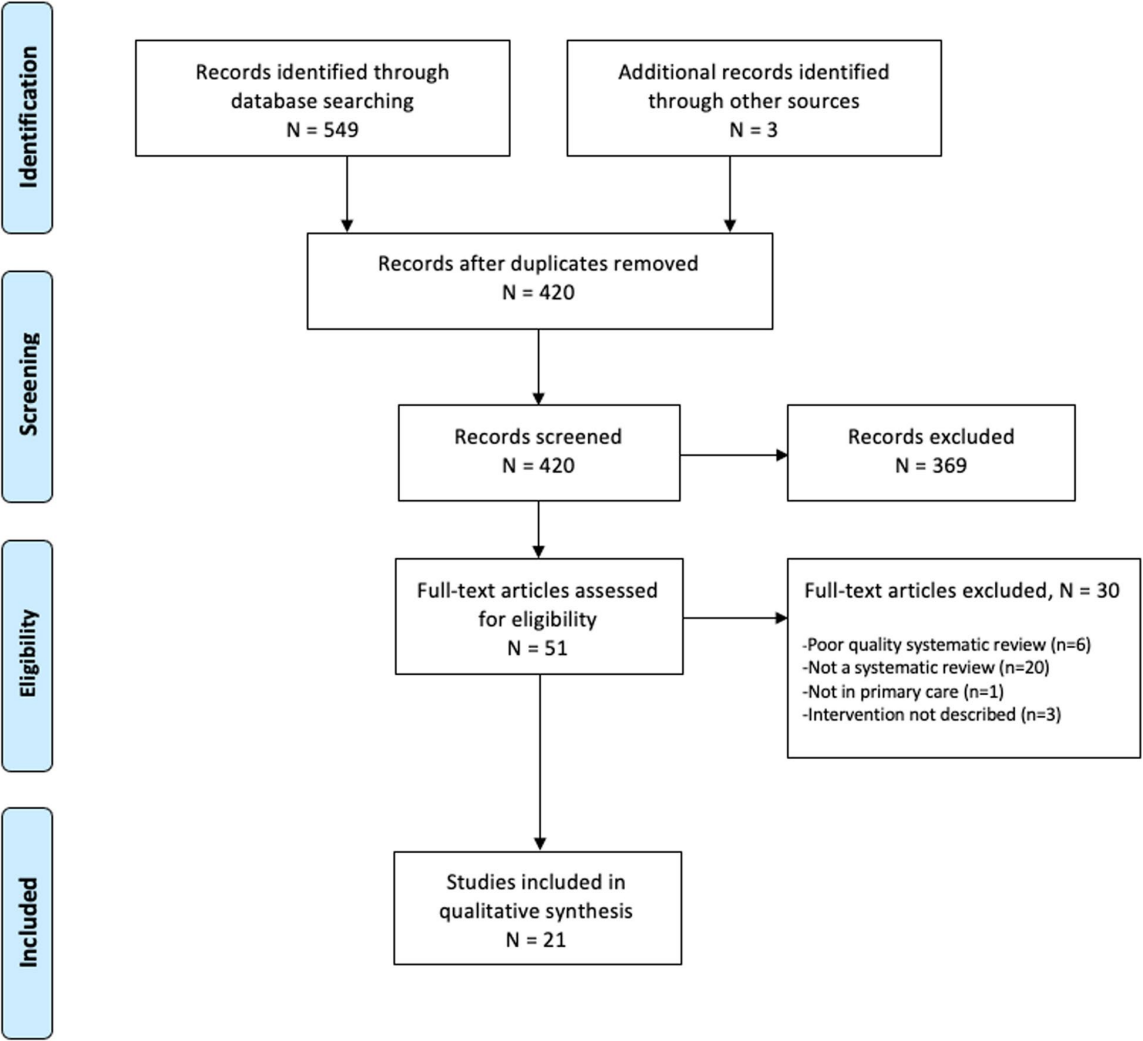
PRISMA flow diagram

The initial search identified 417 unique citations for possible inclusion after duplicates were removed. After searching the reference list of a relevant previous systematic review of systematic reviews [8], three additional citations were collected and screened for eligibility. After screening the 420 citations, 369 were excluded because they did not meet the inclusion criteria. From the 51 full-text articles screened, 30 articles were excluded. Reasons for exclusion include not being a systematic review (*n* = 20), describing a setting other than primary care (*n* = 1), failing to describe the intervention (*n* = 3), or a poor AMSTAR 2 rating (*n* = 6). This resulted in the inclusion of 21 articles (Fig. 1). The included studies were published between June 2003 and July 2019.

### Screening tools

Nine [10–18] out of the 21 included systematic reviews describe screening tools for use in primary care (Table 1). Various screening tools, assessing cognitive impairment or dementia, were compared in terms of cognitive outcomes assessed, time to administer, and sensitivity and specificity. The MMSE was used as a reference standard in the majority of the included studies. The Mini-Cog (*n* = 5) and the MMSE (*n* = 7) were the most widely studied tools among the included reviews. The Mini-Cog takes approximately 3 min to administer, and sensitivity ranges from 76 to 100 % and specificity from 27 to 93 % [10, 12, 14, 17] depending upon the cut-off value used.

**Table 1:** Screening tools and their comparators, cognitive outcomes, administration time, sensitivity and specific and conclusions from the literature included in this systematic review

Reference, Country	Number of studies included in systematic review	Intervention(s)	Comparator	Cognitive outcome(s) measured	Time of administration (minutes)	Sensitivity (%)	Specificity	Conclusions	Abbreviations
Mitchell et al, United Kingdom	44	Multidomain screening tests (known as a battery detection method) in primary care which assess for multiple cognitive domains. Primary care case-finding † : ▪AMTS/MSQ, ▪MSQ ▪WIND-SET ▪PCL ▪AMTS ▪PCL Primary care screening ‡ : ▪ PCL ▪AMTS/MSQ ▪MSQ ▪SPMSQ ▪GPCOG	MMSE	Dementia	Primary care case-finding: ▪AMTS/MSQ = 4 ▪MSQ = 2 ▪WIND-SET = 1 ▪PCL = 11 ▪AMTS = 2 ▪PCL = 11 Primary care screening: ▪PCL = 11 ▪AMTS/MSQ = 4 ▪MSQ = 2 ▪SPMSQ = 2 ▪GPCOG = 5 Comparator: ▪MMSE = 9 with healthy individuals and 15 with patients with dementia.	Battery detection methods: ▪84.0 (95% CI 74.2–91.8)	Battery detection methods: ▪89.9 (95% CI 78.3–97.4)	The optimal individual tools were the AMTS/MSQ and PCL. AMTS was superior to the MMSE for case finding however the MMSE was optimal for screening.	AMTS/MSQ-Abbreviated Mental Test Score/Mental Status Questionnaire, (WIND-SET)-Specific Set of items from MMSE, PCL-Prueba cognitive de leganes, AMTS-Abbreviated mental test score, GPCOG-General practitioner’s assessment of cognition, MMSE-Mini-Mental State Examination † Case-finding is defined as any tool or questionnaire which identifies a condition with minimal false negatives, measured as the positive predicative value. ‡ Screening is the ability of a test to rule out a diagnosis with minimal false positives, reported as the negative predictive value.
Creavin et al, United Kingdom	70	▪MMSE	A commonly accepted clinical (gold) reference standard.	Dementia	▪MMSE=7 with a patient with dementia and 5 with a person with normal cognition	Carnero-Pardo 2013: ▪Cut point of 17 = 70 (95% CI 59-80) ▪Cut point of 24 = 100 (95% CI 95-100)	Carnero-Pardo 2013: ▪Cut point of 17 = 93 (95% CI 89, 96) ▪Cut point of 24 = 46 (95% CI 40-52)	Carnero-Pardo 2013 reported there were some false negatives as the sensitivity fell from 1.00 (95% CI 0.95 to 1.00) to 0.70 (95% CI 0.59 to 0.80). The summary diagnostic accuracy could not be estimated due to insufficient data.	
Abd Razak et al, Malaysia	30	▪MoCA-B; MoCA ▪SPMSQ ▪MEFO ▪ACE-III ▪AQT-CF ▪SLUMS ▪5 Object Test ▪BNB Semantic Fluency ▪SMCC compared to MMSE and CDT ▪CASI-S ▪RCS ▪CPS ▪Literacy Independent Cognitive Assessment ▪BIMS; BCAT ▪3MS ▪Mini-Cog; MIS; MF-2 ▪VT-VSM; VR-DOT ▪CCS ▪CAMCI ▪CADi; CADi-2 ▪DRA ▪p-AD8 ▪IQCODE	Comparing the feasibility and validity between the various screening tools.	Mild cognitive impairment and dementia	▪MoCA-B = 15-21; MoCA = 10-15 ▪SPMSQ = 10-15 ▪MEFO = 10-15 ▪ACE-III = 15 ▪AQT-CF = 3-5 ▪SLUMS = 7 ▪5 Object Test = <5 ▪BNB Semantic Fluency = 31 ▪MCC compared to MMSE and CDT = NR ▪CASI-S = NR ▪RCS = <3 ▪CPS = NR ▪Literacy Independent Cognitive Assessment = 20 ▪BIMS = 3; BCAT = 10-15 ▪3MS = 17 ▪Mini-Cog = 3; MIS = 4; MF-2 = <2 ▪VT-VSM = >12; VR-DOT = NR ▪CCS = 3 ▪CAMCI = 30 ▪CADi = 10; CADi-2 = 10-40 ▪DRA = NR ▪p-AD8 = NR ▪IQCODE = 10	For detecting dementia: ▪ACE-III at a cut-off point of <81, Sn = 100 For detecting MCI: ▪MoCA, Sn = 91-97	For detecting dementia: ▪ACE-III at a cut-off point of <81, Sp=96 For detecting MCI: ▪MoCA, Sp = 60-80	For detecting dementia: Screening tools less sensitive to ACE-III but with relatively high Sn/Sp values were: SLUMS, RCS, and BCAT. For detecting MCI: The MoCA was the most commonly used tool and had the highest Sn/Sp ranges. Less specific to the MoCA but among the most sensitive tools were the (VR-DOT) and IQCODE. Tools with the highest specificity but with lower sensitivity were: The 5 Objects Test, RCS, CPS, and (VT-VSM).	NR-Not Reported, MCI-Mild Cognitive Impairment, (MoCA-B)-Montreal Cognitive Assessment-Basic, (MoCA)-Montreal Cognitive Assessment, SPMSQ-Short Portable Mental Status Questionnaire, (MEFO)-Memory, fluency and orientation, (ACE-III)-Addenbrooke's Cognitive Examination III, (AQT-CF)-A Quick Test of Cognitive Speed, (SLUMS)- Saint Louis University Mental Status, (BNB)-Brief Neuropsychological Battery Semantic Fluency, (SMCC)-The Subjective Memory Complaint Clinical, (CASI-S)-Cognitive Abilities Screening Instrument-Short, (RCS)-Rapid Cognitive Screen, (CPS)-Cognitive Performance Scale, (BIMS)-Brief Interview for Mental Status, (BCAT)-Brief Cognitive Assessment Tool, (3MS)-Modified Mini-Mental State Examination, (MIS)-Memory Impairment Screen, (MF-2)-Memory Function 2, (VT-VSM)-Virtual Reality technology: Virtual supermarket, (VR-DOT)-Virtual Reality Day-Out-Task, (CCS)-Computerized Cognitive Screening Tests, (CAMCI)-Computerized Assessment of Mild Cognitive Impairment, (CADi)-[Cognitive Assessment for Dementia, iPad version], (CADi-2)-[Revised Cognitive Assessment for Dementia, iPad version], (DRA)-Dementia Risk Assessment, (p-AD8)-Participant-rated, (IQCODE)- Informant Questionnaire on Cognitive Decline in the Elderly individuals
Smith et al, United Kingdom	33	▪Rural Older Adult Memory Evaluation ▪Mini-Cog ▪PRISM-PC ▪SAPH questionnaire ▪MMSE and clinical history/examination ▪7-minute screen ▪CIE and MMSE	Not mentioned.	Dementia	Not mentioned.	Not mentioned.	Not mentioned.	There is insufficient evidence to support the adoption of these programmes into practice. Six positive and eight negative effects of primary care screening and early diagnosis of dementia were reported.	(PRISM-PC)-Perceptions Regarding Investigational Screening for Memory in Primary Care, SAPH-Dementia Screening and Perceived Hames, CIE-The Canberra Interview for the Elderly
Brodaty et al, Australia	83	Instruments Validated in General Practice, Community or Population Samples: ▪AMT ▪Cambridge Cognitive Examination ▪CDT ▪GPCOG ▪Mini-Cog ▪MIS ▪MMSE ▪Short and Sweet Screening Instrument ▪Short IQCODE	MMSE	Dementia	▪AMT = 3:16 ▪Camnridge Cognitive Examination = 20 ▪CDT = 2:16 ▪GPCOG = 4.5 ▪Mini-Cog = 2-4 ▪MIS = 4 ▪MMSE = 4 ▪Short and Sweet Screening Instrument = 10 ▪Short IQCODE = 30s	Screening tests validated in general practice, community or population samples: ▪AMT-100 (95% CI 70-100) ▪Cambridge Cognitive Examination-88 (95% CI 64-99) ▪CDT-76 (95% CI 60-88) ▪GPCOG-85 (95% CI 76-92) ▪Mini-Cog-76 (95% CI 65-85) ▪MIS-80 (95% CI 66-90) ▪MMSE-69 (95% CI 66-73) ▪Short and Sweet Screening Instrument-94 (95% CI 88-96) ▪Short IQCODE-79 (95% CI 65-90)	Screening tests validated in general practice, community or population samples: ▪AMT-82 (95% CI 72-90) ▪Cambridge Cognitive Examination-75 (95% CI 67-83) ▪CDT-81 (95% CI 77-84) ▪GPCOG-86 (95% CI 81-91) ▪Mini-Cog-89 (95% CI 87-91) ▪MIS-96 (95% CI 94-98) ▪MMSE-89 (95% CI 87-92) ▪Short and Sweet Screening Instrument-91 (95% CI 90-92) ▪Short IQCODE-82 (95% CI 79-85)	Screening tests validated in general practice, community or population samples: AMT had a PPV=0.42 (95% CI), NPV=1.00 (95% CI), misclassification of 16%, had internal consistency and face validity. Mini-Cog had a PPV=0.34 (95% CI), NPV=0.98 (95% CI), 12% misclassification, no education bias or language/cultural bias, and had face validity*. The AMT, CDT, GPCOG, Short IQCODE, Mini-Cog, and MIS all had a NPV =< MMSE (0.92). The GPCOG, Mini-Cog and MIS had a misclassification rate =< MMSE (15%) and had a high sensitivity and specificity (>=80%) and were therefore chosen as the most suitable instruments for use in general practice.	MAT-Mental Alternation Test. *- (Based on Diagnostic and Statistical Manual of Mental Disorders, Fourth Edition criteria requiring that instruments test memory and at least one other cognitive domain). CDT-Clock Drawing Test. GPCOG-General Practitioner Assessment of Cognition.
Seitz et al, Canada	4	The Mini-Cog performed in insolation or scored based on results on the CDT or three-word recall	Standard diagnostic criteria for the clinical diagnosis of dementia	Alzheimer's disease dementia and related dementias	Mini-Cog = 3-5 in routine practice	Carnero-Pardo 2013 dementia prevalence was 34.5%: ▪100 (95% CI 93-100) Fuchs 2012 5.0% dementia prevalence: ▪100 (95% CI 84-100) Holsinger 2012 (highest quality study) 5.5% dementia prevalence: ▪76 (95% CI 53-92) McCarten 2012 90.3% dementia prevalence: ▪84 (95% CI 81-87)	Carnero-Pardo 2013: ▪40 (95% CI 30-50) Fuchs 2012: ▪85 (95% CI 81-89) Holsinger 2012: ▪73 (95% CI 68-77) McCarten 2012: ▪27 (95% CI 16-41)	Presently there is insufficient evidence to support the use of Mini-Cog in primary care. Studies mentioned are primary journal articles (cross-sectional studies).	
Cullen et al, United Kingdom	36	▪3MS ▪CASI ▪MMSE ▪SASSI ▪STMS ▪CAST ▪GPCOG ▪7MS ▪AMT ▪Mini-Cog ▪SIS ▪T&C ▪ACE-R ▪DemTect	Gold standard diagnostic criteria (based on international diagnostic guidelines or clinical judgement following a full assessment battery).	Cognitive impairment or any type of dementia	▪3MS = 10-15 ▪CASI = 15-20 ▪MMSE = 8-13 ▪SASSI = 10-15 ▪STMS = 5 ▪CAST = 15 ▪GPCOG = 5 ▪7MS = 7-15 ▪AMT = 5 ▪Mini-Cog = 3-4 ▪SIS = 5 ▪T&C = 1 ▪ACE-R = 16 ▪DemTect = 8-10	▪3MS = 83-94 ▪CASI = 91-95 ▪MMSE = 69-91 ▪SASSI = 94 ▪STMS = 86-95 ▪CAST = 88-95 ▪GPCOG = 85 ▪7MS = 91 ▪AMT = 73-100 ▪Mini-Cog = 76-99 ▪SIS = 81-89 ▪T&C = 63-95 ▪ACE-R = 84-94 ▪DemTect = 100 (Alzheimer's dementia)	▪3MS = 85-90 ▪CASI = 37-97 ▪MMSE = 87-99 ▪SASSI = 81-91 ▪STMS = 88-94 ▪CAST = 88-100 ▪GPCOG = 86 ▪7MS = 94 ▪AMT = 71-100 ▪Mini-Cog = 89-93 ▪SIS = 88-91 ▪T&C = 54-96 ▪ACE-R = 89-100 ▪DemTect = 92 (Alzheimer's dementia)	These tests were selected as brief assessment tools in the doctor's office due to their reported sensitivity and specificity values that were >85% for all dementia types together or for more than one particular subtype alone, and/or they covered at least three key domains. The 3MS and CASI are the only tests which cover all six key abilities (Attention/working memory, verbal recall, expressive language, verbal fluency, visual construction, reasoning/judgement).	(ACE-R)-Addenbrooke's Cognitive Examination Revised, STMS-Short Test of Mental Status, CCSE-Cognitive Capacity Screening Examination, (R-CAMCOG)-Rotterdam Version of the Cambridge Cognitive Examination
Lischka et al, Canada	12	▪MIS ▪IST, BVRT ▪CAMCI ▪ACE ▪ADAS-Cog ▪CAMCOG ▪MoCA ▪S-MMSE ▪IQCODE ▪STMS ▪MMSE ▪HDS-R ▪CCSE	A full clinical examination as the reference standard.	Dementia, MCI, amnestic MCI, mild dementia, and questionable dementia.	▪MIS, IST = 4 ▪IST, BVRT = 1 ▪CAMCI = 15 ▪ACE = 15 ▪ADAS-Cog = NR ▪CAMCOG = 20 ▪MoCA = 10-12 ▪S-MMSE = 10 ▪IQCODE = 10-20 ▪STMS = 5 ▪MMSE = 5-10 ▪HDS-R = NR ▪CCSE = 10-12	▪MIS, IST = 74 ▪IST, BVRT - Cutoff level 1 = 90.8 ▪CAMCI = 83.4 ▪ACE - Cutoff <88/100 = 100 ▪ADAS-Cog - Cutoff <75/100 = 85 ▪CAMCOG = 76 for memory section ▪MoCA = 94 ▪S-MMSE = 14 ▪IQCODE = 41 ▪STMS = ≤ 80 ▪MMSE = 31 ▪HDS-R = 92 for the dementia diabetic group ▪CCSE - Cutoff 26/25 = 88.1	▪MIS = 84, IST = 81 ▪IST, BVRT - Cutoff level 1 = 52.2 ▪CAMCI = 78.5 ▪ACE - Cutoff <88/100 = 43 ▪ADAS-Cog - Cutoff <75/100 = 83 ▪CAMCOG = 96 for memory section ▪MoCA = 50 ▪S-MMSE = 100 ▪IQCODE = 67 ▪STMS = ≤ 80 ▪MMSE = 96 ▪HDS-R = 74 for the dementia diabetic group ▪CCSE - Cutoff 26/25 = 83.5	Tools with the highest specificity rates: ▪MMSE ▪S-MMSE Tests with the highest sensitivities: ▪HDS-R ▪ACE, which decreased depending on cut-off value ▪MoCA for the dementia group and 83% for the MCI group ▪CAMCI ▪CCSE ▪The combination of the MMSE, IST, and BVRT at 90.8% for the first cut-off level. The ACE demonstrated good diagnostic accuracy with AUC=0.98. Xu et al. (2002) found that the CCSE was the best predictive screen in MCI participants for diagnosing all dementia due to its high sensitivity (88.1%) and specificity (83.5%).	(IST,BVRT)-Isaacs Set Test, Benton's Visual Retention Test. CAMCI-Chinese Abbreviated Mild Cognitive Impairment Test, (ADAS-Cog)-Alzheimer Disease Assessment Scale-Cognitive Subscale, (S-MMSE)-Standardized Mini-Mental State Examination, (HDS-R)-Hasegawa Dementia Scale-Revised, CCSE-Cognitive Capacity Screening Examination, CAMCOG-Cambridge Cognitive Examination
Boustani et al, United States	61	▪MMSE ▪FAQ ▪BIMC ▪BOMC ▪STMS	DSM-IV	Dementia	Not mentioned.	▪MMSE = 71-92 ▪FAQ = 90 ▪BIMC = 90 ▪BOMC = 69 ▪STMS = 81	▪MMSE = 56-96 ▪FAQ = 90 ▪BIMC = 65-90 ▪BOMC = 90 ▪STMS = 90	The MMSE has limited Sp when the cut-point is set for higher Sn. Accuracy of the MMSE changes based upon the patients age, education level and ethnicity and therefore requires adjustment when used.	BIMC-Blessed Information Memory Concentration; BOMC-Blessed Orientation Memory Concentration; FAQ-Functional Activities Questionnaire; STMS-Short Test of Mental Status; DSM-IV-Diagnostic and Statistical Manual of Mental Disorders, fourth edition

Five systematic reviews examining the MMSE found that it took between 4 and 15 min to administer depending upon the severity of dementia [12–16]. One study found a cut point of 17 had a higher specificity (93 %, 95 % CI: 89-96 %) than a cut point of 24 (46 %, 95 % CI: 40-52 %), while the sensitivity fell from 100 % (95 % CI: 95-100 %) to 70 % (95 % CI: 59-80 %) respectively [16].

The Abbreviated Mental Test Score (AMTS) achieved high sensitivity (100 %, 95 % CI: 70-100 %) and specificity (82 %, 95 % CI: 72-90 %) [12] compared to a clinical reference standard, and took the shortest amount of time (3.16 to 5 min) [12, 14] within primary care. The AMTS was validated for use in general practice [12].

### Diagnostic accuracy and physician education

The diagnosis of dementia by FPs varies but is generally low, as reported in 3 different systematic reviews [11, 16, 19]. In an (urban/rural) study, when following usual practice, only half of cases of mild dementia were diagnosed by the FP [19]. In a separate review, un-diagnosed dementia accounted for 50 − 66 % of all cases of dementia in three primary care samples studied [11, 20−22]. Another review reported that the recognition of cognitive impairment in usual practice achieved a detection sensitivity of 62.8 % (95 % CI: 38.0-84.4 %) and specificity of 87.3 % (*n* = 3; 95 % CI: 84.9-89.4 %) [16]. However, medical record notations mentioning dementia were present in only 37.9 % (95 % CI: 26.8-49.6 %) and FPs recorded a definitive dementia diagnosis in the medical record in only 10.9 % (95 % CI: 6.8-15.7 %) of mild cognitive impairment (MCI) cases [16].

Five of six studies found that FPs had an increased likelihood of suspecting dementia after attending an educational seminar [23, 24]. One study found that the length of the educational seminar impacted the degree of knowledge about dementia management [24].

### Management of dementia

Decision aids, advanced care planning (ACP), collaboration with a case manager (CM) and practice guidelines are all interventions with variable impact on helping facilitate the management of dementia in primary care [23, 25−29] (Table 2). A CM in particular, such as a nurse specialized in care of older adults, can be an asset to a primary care team with the collective goal of collaborating towards meeting the needs of the patient-caregiver dyad [30]. In the case management intervention group of a randomized controlled trial, neuropsychiatric symptoms of dementia decreased (Mean Effect Size (MES) = 0.88), as well as the numbers of hospital (MES = 0.66) and emergency department admissions (MES = 0.17) [26]. However, it was found that there was a lack of successful implementation of a CM into care teams within primary care because of the absence of CMs within the primary care setting, and 52 % of CMs reported ineffective communication between the CM and FPs [26].

**Table 2:** Case management interventions and corresponding comparators and outcomes from the literature included in this systematic review

Authors, Country	Number of studies included in systematic review	Intervention	Comparator	Outcomes
Sivananthan et al, Canada	12	7 dementia care processes recommended by best practice guidelines: ▪Formal memory testing ▪Imaging ▪Laboratory testing ▪Interventions ▪Counseling ▪Community service ▪Specialist referrals	Clinical services provided by physicians to older adults diagnosed with dementia.	▪8 out of 12 studies reported that <60% of physicians conducted formal memory testing, while 3 studies reported <15%, and 1 study <4% ▪33% to 91% of family physician's prescribed medications for dementia and consequent behavioral problems ▪33-80% of physicians reported the use of CT or MRI as a diagnostic tool, and >75% used blood work ▪2 studies reported that >80% of physicians provided counseling.
Khanassov et al, Canada	23	Case Management interventions comprising all components identified by the Case Management Society of America: ▪Case finding and screening ▪Assessment ▪Care planning ▪Implementation and management ▪Monitoring ▪Review	No comparator	▪Only 63% of case managers clearly explained their role to the patient-caregiver dyads while 25% did not give any detail during assessment ▪52% of case managers indicated that poor communication with healthcare providers negatively affected their work ▪Limiting factors to case management implementation were: insufficient knowledge of diagnostic tools, absence of training, and the absence of the case manager in the primary care setting.
Davies et al, United Kingdom	10	Decision-making interventions with decision aids in dementia care (i.e. audio guided booklet, a printed decision aids about dementia and feeding; a living with dementia Guiding Options for Living with Dementia (GOLD) book; DECIDE intervention: a guided decision aid participants read and complete with support of decision coach to assist in making decisions regarding care home placement, video decision aid and structured meeting between surrogate decision maker and interdisciplinary care plan team; a video decision aid and audio description of advanced dementia)	▪The majority of studies used a control group ▪One study used solely listening to a verbal narrative of the disease.	Place of care: ▪DECIDE decreased decisional conflict in caregivers ▪GOLD showed less of an increase in burden and greater increase in the knowledge of caregivers Goals of care: ▪A video decision aid combined with a structured meeting improved communication between caregivers and professionals and improved the concordance on the goals of care after 9 months Meta-analysis: ▪Two RCTs (N=72) included. ▪Decision aids are effective in decreasing decisional conflict in caregivers (standardized MD=− 0.50, 95% CI [ − 0.97, − 0.02]). This suggests increased confidence in decision-making and understanding of the decisions. ▪Decisional conflict was measured using the Decision Conflict Scale at 3 months post intervention.
Tilburgs et al, Australia	16	Advanced care planning (ACP)	No comparator	Facilitators for ACP: ▪An early start while cognitive decline is mild. ▪Inclusion of all stakeholders and a good relationship between the GP, patient, and family carers. ▪Discussion of social and medical issues aimed at maintaining a normal life. ▪Decision aids that provide information and structure which contribute to decision making. Barriers for ACP: ▪Uncertainty about the timing of ACP. ▪How to plan for an uncertain future. ▪Lack of knowledge about dementia and patient's lack of knowledge of diagnosis. ▪Bad relationships among stakeholders. ▪Stress/fear caused by ACP. ▪Who should take initiative for ACP. ▪Difficulties assessing the dementia patient's decisional capacities. ▪Changing preferences.
Mukadam et al, United Kingdom	13	Interventions intended to increase the detection of : ▪Dementia ▪Suspected dementia ▪People presenting with memory complaints	RCT: ▪Control groups. Non-randomized studies and pre-post study designs: ▪Comparison groups.	▪2 of 3 RCTs of physician education found group educational interventions increased the likelihood of physicians suspecting dementia. ▪Non-randomized study findings suggest that clinician education in primary care interventions can increase the proportion of patients in whom physicians suspect dementia; untargeted community leaflet campaigns did not increase dementia diagnosis rates. ▪Pre-post comparison studies showed no positive effects for individual clinician training, group training with a routine screening programme or a targeted leaflet campaign. An increased number of memory clinics correlated with an increased number of dementia diagnoses.
Khanassov et al, Canada	43	Case management (CM): ▪Assessment ▪Care planning ▪Implementation ▪Management ▪Regular follow-up	RCT: ▪Control group Qualitative studies: ▪No control	RCT evidence: ▪4/10 trials showed a decrease in the frequency of behavioral symptoms of dementia in the CM intervention group (mean effect size 0.88), while 2/7 reported a decrease in depression symptoms. ▪No effect on cognition and perceived health was observed. ▪8/11 trials found no effect on institutionalization. ▪Hospital admissions decreased (MES=0.66) in 2/5 studies. ▪Decreased ER admission was observed in 1/3 studies (effect size: 0.17) and a decrease in length of hospital stay was shown in both of the studies that evaluated this outcome (MES=1.06). ▪For caregivers, 5/10 studies showed a decrease in depression (MES=0.68) and 4/11 showed a decrease in burden (MES=0.5). Barriers to implementation of CM using outcome matching: ▪Intervention durations being too short. ▪Need for high-intensity CM. ▪Scarce communication. ▪Case manager and physician in different locations. ▪Lack of healthcare providers with geriatric training. Addressing these barriers correlated with better outcomes, as studies addressing more barriers resulted in more positive outcomes (agreement κ=0.94; CI, 0.82-1.1).
Perry et al, Netherlands	6	Series of seminars and the appointment of dementia care managers.	Control groups in studies: ▪Clinical practice guidelines for dementia received by mail ▪No training ▪No seminars ▪No training and no dementia care managers ▪Short, partly interactive seminar on dementia diagnostics (3 hours).	▪Intervention clinics demonstrated better health-related quality of life (QoL), overall quality of health care in patients, family caregiving quality, social support and more family caregivers reported receiving all the help they needed. ▪The health-related QoL of the caregiver did not increase. ▪Higher proportions of patients were newly diagnosed with dementia following educational workshops and computerized Decision Support System (DSS) group compared to the control group. ▪After a 2-h seminar for physicians there were higher rates of 'suspected dementia' and lower rates of both 'uncertain' and 'non-suspected' diagnoses when compared to the control group. ▪Both the mean compliance per patient to the total set of 23 quality indicators, and the compliance per indicator for 21 of 23 quality indicators, were better in intervention clinics than in control clinics. ▪Physicians gained more knowledge after a 5-h seminar than a 3-h seminar. ▪After 9-months, more physicians in the intervention group correctly answered 2 questions about decision-making compared to the control group. Those in the intervention group more strongly agreed that 'Older patients with dementia are difficult to manage in primary care' than the PCPs in the control group.

Only one systematic review looked at pharmacological treatments in the context of primary care [11]. There was no clinically important difference observed on neuropsychiatric symptoms between patients with mild to moderate Alzheimer’s disease taking cholinesterase inhibitors versus placebo [11].

### Supporting caregivers of people with dementia

FPs reported feeling highly involved in dementia care [31]. However, family caregivers reported that communication with the FPs was unsatisfactory, specifically around awareness of daily care problems (e.g. neuropsychiatric symptoms) [31]. The primary care educational intervention, Resources for Enhancing Alzheimer’s Caregiver Health (Department of Veterans Affairs) (REACH VA), involves a trained coach who provides sessions to the caregiver on topics relating to self-care, problem solving, mood management and stress management [32]. REACH VA was successful at increasing carer ability to manage problem behaviours and improved outcomes for caregivers, such as decreased burden, depression and caregiving frustrations [30, 31]. A meta-analysis showed that 58 % (95 % CI: 43-72 %) of family caregivers were in favor of early dementia diagnosis, 50 % (95 % CI: 35-65 %) needed education on dementia, and 23 % (95 % CI: 17-31 %) needed in-home support [33].

## Discussion

This systematic review of systematic reviews identified evidence to inform processes for diagnosis and management of dementia within primary care. While the diagnostic accuracy of a tool may be high, the time taken to administer the tool and copyright limitation for tool use are also important to consider in the context of a busy primary care office. The MMSE, which is copyrighted, may not be the best test for use in general practice. Instead, the AMTS appears to be the most suitable tool for use in a busy primary care office, as it has good diagnostic accuracy, does not appear to be copyright protected and takes less time to administer than the MMSE [12, 14, 15]. The Mini-Cog is also quick to administer, and a Cochrane systematic review evaluating the Mini-Cog across care settings recommended that the Mini-Cog be used initially as a case finding test to identify patients who would benefit from additional cognitive evaluations for dementia [34]. However, the sensitivity of the Mini-Cog may not be high enough to be considered useful in primary care [17], as too many cases would be missed.

The current literature suggests that the implementation of case management directly into the primary care setting can be of great benefit to the patient-caregiver dyad, as well as to the health care system. The CM can help facilitate the advanced care planning process [29], as well as decrease the frequency of neuropsychiatric symptoms of dementia, symptoms of depression, hospital admissions and length of stay in hospital; caregivers can also benefit by experiencing decreased burden and depression [26]. A Cochrane review evaluating the effectiveness of case management in community settings lends support to dementia case management, finding that carer burden decreased and fewer patients where institutionalized after 6 months [35]. Further, there was a reduction in residential home and hospital use after 6 months of case management implementation [35]. There is however a lack of evidence related to cost effectiveness of case management. Facilitating successful case management and advanced care planning includes early implementation while cognitive decline is mild, involving all stakeholders (caregiver, patient, family and FP), and fostering a good relationship between the FP and patient-caregiver dyad [29]. The CM should be physically present in the primary care setting, clearly explain their role to all stakeholders, implement high-intensity case management, and communicate frequently to all stakeholders in order to ensure positive outcomes for the patient-caregiver dyad [26, 27].

Combining educational seminars for FPs with dementia case management may be the best management strategy [23, 24]. Educational interventions focused on dementia diagnosis and management in the context of primary care increased the likelihood of FPs suspecting dementia, while also improving the experience of the family caregiver and the patient [23, 24].

There was limited evidence concerning the use of pharmacological interventions for the treatment of dementia within the primary care setting. Unfortunately, many pharmacologic studies do not focus on primary care or FPs, making it difficult to draw conclusions about the approach to take regarding the use of medications in this context. One systematic review found no clinically important differences between groups receiving cholinesterase inhibitors and those receiving a placebo in the development of behavioral and neuropsychiatric symptoms of Alzheimer’s disease [11]. Similarly, cholinesterase inhibitor use was found to have uncertain clinical benefit in a recent systematic review that explored the benefits and harms of prescription drugs for the treatment of Alzheimer disease, regardless of care setting [36]. This recent review also found limited benefit for memantine.

## Conclusions

The AMTS is suitable for detecting dementia within primary care given its high sensitivity and short administration time. To improve dementia identification, FPs should participate in educational interventions. Incorporation of CMs into the primary care team can help with dementia management and result in improved outcomes. There is limited evidence supporting the benefit for pharmacological treatments in the context of primary care.

### Limitations and Future Research

A limitation of this systematic review of systematic reviews includes the exclusion of possibly relevant pharmacological reviews, given the fact that we focused on studies conducted in the primary care setting. Future pharmacological studies conducted in the specific context of primary care are needed. Additionally, the results from our review are limited to literature from countries that clearly distinguish primary care from specialist care, given the focus of the search strategy. Lastly, many of the studies included within the identified systematic reviews inappropriately used the MMSE as a reference tool when determining the sensitivity and specificity of various screening tools. Further studies should compare commonly used screening tools within primary care to a recognized gold standard.

## Supplementary Information

**Additional file 1.**

## Data Availability

All data generated or analysed during this study are included in this published article in Additional file 1: Appendixes 1 and 2.

## Abbreviations

FPs: Family physicians
*PRISMA*: Preferred Reporting Items for Systematic Reviews and Meta-analyses
MMSE: Mini-Mental State Examination
AMTS: Abbreviated Mental Test Score
ACP: Advanced Care Planning
CM: Case Manager
MES: Mean Effect Size
REACH VA: Resources for Enhancing Alzheimer’s Caregiver Health (Department of Veterans Affairs)
