# Supplementary material for: Optimizing the diagnosis and management of dementia within primary care: a systematic review of systematic reviews
Source: BMC Fam Pract. 2021 Aug 11;22:166. doi: 10.1186/s12875-021-01461-5 (PMC8359121; doi:10.1186/s12875-021-01461-5)
Supplement: Supplementary file 1 — Additional file 1. [file 12875_2021_1461_MOESM1_ESM.zip › AMSTAR 2 Supplementary Information Appendix 3.pdf]

Title of Reference	Authors	1. Did the research questions and inclusion criteria for the review include the components of PICO?	2. Did the report of the review contain an explicit statement that the review methods were established prior to the conduct of the review and did the report justify any significant deviations from the protocol?	3. Did the review authors explain their selection of the study designs for inclusion in the review?	4. Did the review authors use a comprehensive literature search strategy?	5. Did the review authors perform study selection in duplicate?	6. Did the review authors perform data extraction in duplicate?	7. Did the review authors provide a list of excluded studies and justify the exclusions?	8. Did the review authors describe the included studies in adequate detail?	9. RCTs - Did the review authors use a satisfactory technique for assessing the risk of bias (RoB) in individual studies that were included in the review?	9. NRSI - Did the review authors use a satisfactory technique for assessing the risk of bias (RoB) in individual studies that were included in the review?	10. Did the review authors report on the sources of funding for the studies included in the review?	11. RCTs - If meta-analysis was performed did the review authors use appropriate methods for statistical combination of results?	11. NRSI - If meta-analysis was performed did the review authors use appropriate methods for statistical combination of results?	12. If meta-analysis was performed, did the review authors assess the potential impact of RoB in individual studies on the results of the meta-analysis or other evidence synthesis?	13. Did the review authors account for RoB in individual studies when interpreting/discussing the results of the review?	14. Did the review authors provide a satisfactory explanation for, and discussion of, any heterogeneity observed in the results of the review?	15. If they performed quantitative synthesis did the review authors carry out an adequate investigation of publication bias (small study bias) and discuss its likely impact on the results of the review?	16. Did the review authors report any potential sources of conflict of interest, including any funding they received for conducting the review?
Screening and Case Finding Tools for the Detection of Dementia. Part I: Evidence- Based Meta-Analysis of Multidomain Tests	Alex J. Mitchell, M.R.C.Psych., Srinivasa Malladi, M.R.C.Psych.	Yes	No	Yes	No	No	No	No	Partial Yes	No	No	No	No	No	Yes	No	Yes	Yes	No
Mini-Mental State Examination (MMSE) for the detection of dementia in clinically unevaluated people aged 65 and over in community and primary care populations (Review)	Creavin ST, Wisniewski S, Noel-Storr AH, Trevelyan CM, Hampton T, Rayment D, Thom VM, Nash KJE, Elhamoui H, Milligan R, Patel AS, Tsivos DV, Wing T, Phillips E, Kellman SM, Shackleton HL, Singleton GF, Neale BE, Watton ME, Cullum S	Yes	Yes	Yes	Yes	Yes	Yes	Yes	Yes	No	Includes only RCTs	No	Yes	No	Yes	Yes	Yes	Yes	Yes
Validity of screening tools for dementia and mild cognitive impairment among the elderly in primary health care: a systematic review	M.A. Abd Razak, N.A. Ahmad, Y.Y. Chan, N. Mohamad Kasim, M. Yusof, M.K.A. Abdul Ghani, M. Omar, F.A. Abd Aziz, R. Jamaluddin	Yes	No	No	Partial Yes	Yes	Yes	No	No	No	No	No	No meta-analysis conducted	No meta-analysis conducted	No meta-analysis conducted	No	No	No meta-analysis conducted	Yes
Systematic Review Investigating Multi-disciplinary Team Approaches to Screening and Early Diagnosis of Dementia in Primary Care – What are the Positive and Negative Effects and Who Should Deliver It?	Toby Smith, Jane Cross, Fiona Poland, Felix Clay, Abbey Brookes, Ian Maidment, Bridget Penhale, Ken Laidlaw and Chris Fox.	Yes	Yes	Yes	Partial Yes	Yes	Yes	No	Partial Yes	No	No	No	No meta-analysis conducted	No meta-analysis conducted	No meta-analysis conducted	No	No	No meta-analysis conducted	Yes

What Is the Best Dementia Screening Instrument for General Practitioners to Use?	Henry Brodaty, M.B.B.S., M.D., F.R.A.C.P., F.R.A.N.Z.C.P., Lee-Fay Low, B.Sc.(Psych.)Hons., Louisa Gibson, B.Sc.(Arch.), Grad. Dip. Psych., B.Sc.(Psych.)Hons., Kim Burns, R.N., B.Psych.(Hons.)	Yes	No	Yes	Partial Yes	No	No	No	No	No	No	No	No meta-analysis conducted	No meta-analysis conducted	No meta-analysis conducted	Yes	No	No meta-analysis conducted	No
Mini-Cog for the diagnosis of Alzheimer's disease dementia and other dementias within a primary care setting (Review)	Seitz DP, Chan CCH, Newton HT, Gill SS, Herrmann N, Smailagic N, Nikolaou V, Fage BA	Yes	No	Yes	Partial Yes	Yes	Yes	Yes	No	No	Includes only RCTs	No	No meta-analysis conducted	No meta-analysis conducted	No meta-analysis conducted	Yes	Yes	No meta-analysis conducted	Yes
A review of screening tests for cognitive impairment	Breda Cullen, Brian O'Neill, Jonathan J Evans, Robert F Coen, Brian A Lawlor	Yes	No	No	Partial Yes	No	Yes	No	No	No	No	No	No meta-analysis conducted	No meta-analysis conducted	No meta-analysis conducted	No	No	No meta-analysis conducted	Yes
A Systematic Review of Screening Tools for Predicting the Development of Dementia	Andrea R. Lischka, Marissa Mendelsohn, and Tom Overend	Yes	Partial Yes	Yes	Partial Yes	Yes	No	No	No	No	No	No	No meta-analysis conducted	No meta-analysis conducted	No meta-analysis conducted	Yes	No	No meta-analysis conducted	No
Screening for Dementia in Primary Care: A Summary of the Evidence for the U.S. Preventive Services Task Force	Malaz Boustani, MD, MPH; Britt Peterson, MD, MPH; Laura Hanson, MD, MPH; Russell Harris, MD, MPH; and Kathleen N. Lohr, PhD	Yes	No	Yes	Partial Yes	Yes	Yes	No	No	No	Includes only RCTs	No	No meta-analysis conducted	No meta-analysis conducted	No meta-analysis conducted	No	No	No meta-analysis conducted	No
Variations in Self-Reported Practice of Physicians Providing Clinical Care to Individuals with Dementia: A Systematic Review	Saskia N. Sivananthan, MSc, Joseph H. Puyat, MA, MSc, and Kimberlyn M. McGrail, PhD	Yes	Partial Yes	Yes	Partial Yes	Yes	Yes	No	Partial Yes	Includes only NRSI	No	No	No meta-analysis conducted	No meta-analysis conducted	No meta-analysis conducted	Yes	Yes	No meta-analysis conducted	Yes
Case management for dementia in primary health care: A systematic mixed studies review based on the diffusion of innovation model	Khanassov V., Vedel I., Pluye P.	Yes	No	Yes	Partial Yes	Yes	Yes	No	No	No	No	No	No meta-analysis conducted	No meta-analysis conducted	No meta-analysis conducted	No	No	No meta-analysis conducted	Yes
Decision aids to support decision-making in dementia care: a systematic review	Nathan Davies, Brooke Schiowitz, Greta Rait, Victoria Vickerstaff, and Elizabeth L. Sampson	Yes	Yes	No	Partial Yes	Yes	Yes	No	Partial Yes	No	Includes only RCTs	No	No	No	No	No	Yes	No	Yes

Barriers and facilitators for GPs in dementia advance care planning: A systematic integrative review	Bram Tilburgs, Myrra Vernooij-Dassen, Raymond Koopmans, Hans van Gennip, Yvonne Engels, Marieke Perry	Yes	Partial Yes	Yes	Partial Yes	Yes	Yes	No	Partial Yes	No	No	No	No meta-analysis conducted	No meta-analysis conducted	No meta-analysis conducted	No	No	No meta-analysis conducted	Yes
A systematic review of interventions to detect dementia or cognitive impairment	Naaheed Mukadam, Claudia Cooper, Nishin Kherani and Gill Livingston	Yes	No	Yes	Yes	Yes	Yes	No	Yes	No	No	No	No meta-analysis conducted	No meta-analysis conducted	No meta-analysis conducted	No	No	No meta-analysis conducted	Yes
Barriers to Implementation of Case Management for Patients With Dementia: A Systematic Mixed Studies Review	Vladimir Khanassov, MD Isabelle Vedel, MD, PhD, Pierre Pluye, MD, PhD	Yes	No	Yes	Partial Yes	Yes	Yes	No	Yes	No	No	No	No meta-analysis conducted	No meta-analysis conducted	No meta-analysis conducted	No	No	No meta-analysis conducted	Yes
Effects of educational interventions on primary dementia care: A systematic review	M. Perry, I. Draškovic,́ P. Lucassen, M. Vernooij-Dassen, T. van Achterberg and M. Olde Rikkert	Yes	No	Yes	Partial Yes	Yes	Yes	No	Yes	Partial Yes	Includes only RCTs	No	No meta-analysis conducted	No meta-analysis conducted	No meta-analysis conducted	No	No	No meta-analysis conducted	Yes
What is the role of the general practitioner towards the family caregiver of a community-dwelling demented relative?	Birgitte Schoenmakers, Frank Buntinx & Jan Delepeleire	Yes	No	No	Yes	No	No	No	No	No	Includes only RCTs	No	No meta-analysis conducted	No meta-analysis conducted	No meta-analysis conducted	No	No	No meta-analysis conducted	No
General practice based psychosocial interventions for supporting carers of people with dementia or stroke: a systematic review	Nan Greenwood , Ferruccio Pelone and Anne-Marie Hassenkamp	Yes	Partial Yes	Yes	Partial Yes	Yes	Yes	Yes	Yes	Yes	Includes only RCTs	No	No meta-analysis conducted	No meta-analysis conducted	No meta-analysis conducted	No	No	No meta-analysis conducted	Yes
Family Physician–Case Manager Collaboration and Needs of Patients With Dementia and Their Caregivers: A Systematic Mixed Studies Review	Vladimir Khanassov, MD, MSc Isabelle Vedel, MD, PhD	Yes	No	Yes	Partial Yes	Yes	Yes	Partial Yes	No	No	No	No	Yes	No	No	No	No	No	Yes
The accuracy of family physicians' dementia diagnoses at different stages of dementia: a systematic review	Pim van den Dungen, Harm W. M. van Marwijk, Henriëtte E. van der Horst, Eric P. Moll van Charante, Janet MacNeil Vroomen, Peter M. van de Ven and Hein P. J. van Hout	Yes	No	Yes	Partial Yes	Yes	Yes	No	No	No	No	No	No meta-analysis conducted	No meta-analysis conducted	No meta-analysis conducted	Yes	Yes	No meta-analysis conducted	Yes

Clinical recognition of dementia and cognitive impairment in primary care: a meta-analysis of physician accuracy	Mitchell AJ, Meader N, Pentzek M.	Yes	No	No	Partial Yes	No	Yes	No	Partial Yes	No	No	No	No	No	No	No	Yes	Yes	Yes
