# Supplementary material for: Optimizing the diagnosis and management of dementia within primary care: a systematic review of systematic reviews
Source: BMC Fam Pract. 2021 Aug 11;22:166. doi: 10.1186/s12875-021-01461-5 (PMC8359121; doi:10.1186/s12875-021-01461-5)
Supplement: Supplementary file 1 — Additional file 1. [file 12875_2021_1461_MOESM1_ESM.zip › Search Stategy-Supplementary Information Appendix 1.pdf]

Optimizing the Diagnosis and Management of Dementia within Primary Care: A Systematic Review of Systematic Reviews

Supplementary Information Appendixes 1, 2 and 3

Brooklynn Fernandes[1], Zahra Goodarzi MD MSc[2], Jayna Holroyd-Leduc MD[2]

1. Undergraduate Student, Faculty of Science, University of Calgary
2. Departments of Medicine and Community Health Sciences, Cumming School of Medicine, University of Calgary

Primary care/Family medicine	Dementia	Systematic reviews
Primary health care/<br><br>Physicians, primary care/<br>General practitioner/<br><br>Physicians, Primary Care/ed [Education]<br><br>((primary care or primary health care or primary healthcare or primary or general practice or general) adj2 (doctor* or physician* or practitioner* or practice*)).kf,tw<br><br>Family practice/<br>General practice/<br><br>((family or general) adj2 (practice* or medicine or doctor* or physician* or practitioner* or practice physician) or physicians, family or general medicine or general medical practice*).kw,tw	Dementia/<br><br>Dementia/px [Psychology]<br>dementia*.kf,tw<br><br>Dementia, Multi-Infarct/<br><br>AIDS Dementia Complex/<br><br>Lewy Bodies/<br>Lewy Bod*.mp<br><br>Cognition disorders/<br><br>Cognition disorders/di [Diagnosis]<br><br>(Cognition disorder* or disorder*, cognition or (cognitive adj2 (disorder* or defect* or disability or dysfunction or impairment))).kf,tw<br><br>Memory Disorders/<br>(Memory disorder* or age related memory disorder* or memory deficit* or memory disorder*, age related or memory defect or memory impairment*).kf,tw<br><br>Neurodegenerative Diseases/<br>(Neurodegenerative disease* or degenerative condition*, neurologic or degenerative neurological disease* or degenerative neurological disorder* or neurologic degenerative condition*).mp<br><br>Alzheimer Disease/<br>((Alzheimer adj2 (disease* or syndrome or dementia*)) or Alzheimer disease, early onset or Alzheimer disease, late onset or Alzheimer type dementia or Alzheimer type senile dementia or Alzheimer's disease* or	CADTH search for systematic reviews<br><br>Limit to English language<br><br>Systematic Reviews/Meta-Analysis/Health Technology Assessment – OVID Medline, Embase, PsycINFO<br>1. meta-analysis.pt.<br><br>2. meta-analysis/ or systematic review/ or meta-analysis as topic/ or "meta analysis (topic)"/ or "systematic review (topic)"/ or exp technology assessment, biomedical/<br><br>3. ((systematic* adj3 (review* or overview*)) or (methodologic* adj3 (review* or overview*))).ti,ab,kf,kw.<br><br>4. ((quantitative adj3 (review* or overview* or synthes*)) or (research adj3 (integrati* or overview*))).ti,ab,kf,kw.<br><br>5. ((integrative adj3 (review* or overview*)) or (collaborative adj3 (review* or overview*)) or (pool* adj3 analy*)).ti,ab,kf,kw.<br><br>6. (data synthes* or data extraction* or data abstraction*).ti,ab,kf,kw.<br><br>7. (handsearch* or hand search*).ti,ab,kf,kw.<br><br>8. (mantel haenszel or peto or der simonian or dersimonian or fixed effect* or latin square*).ti,ab,kf,kw.<br><br>9. (met analy* or metanaly* or technology assessment* or HTA or HTAs or technology overview* or technology appraisal*).ti,ab,kf,kw.<br><br>10. (meta regression* or metaregression*).ti,ab,kf,kw.<br><br>11. (meta-analy* or metaanaly* or systematic review* or biomedical technology assessment* or bio-medical technology assessment*).mp,hw.<br><br>12. (medline or cochrane or pubmed or medlars or embase or cinahl).ti,ab,hw.

	dementia, Alzheimer or early onset Alzheimer disease* or late onset Alzheimer disease* or dementia, Alzheimer).mp<br><br>Neurocognitive Disorders/ (Neurocognitive disorder* or neurocognitive disease*).mp	13. (cochrane or (health adj2 technology assessment) or evidence report).jw.<br><br>14. (meta-analysis or systematic review).md.<br><br>15. (comparative adj3 (efficacy or effectiveness)).ti,ab,kf,kw.<br><br>16. (outcomes research or relative effectiveness).ti,ab,kf,kw.<br><br>17. ((indirect or indirect treatment or mixed-treatment) adj comparison*).ti,ab,kf,kw.<br><br>18. or/1-17

Database: Ovid MEDLINE(R) and Epub Ahead of Print, In-Process & Other Non-Indexed Citations and Daily <1946 to October 01, 2020>

Search Strategy:

----

1 Primary Health Care/ (78241)

2 Physicians, primary care/ (3534)

3 Physicians, Primary Care/ed [Education] (451)

4 ((primary care or primary health care or primary healthcare or primary) adj2 (doctor* or physician* or practitioner*)).kf,tw. (25959)

5 general practice/ or family practice/ (75156)

6 (family practice* or general practice* or family medicine or family doctor* or family physician*).kf,tw. (75746)

7 or/1-6 (198484)

8 Dementia/ (51505)

9 Dementia/px [Psychology] (11404)

10 dementia*.kf,tw. (115272)

11 Dementia, Multi-Infarct/ (1089)

12 AIDS Dementia Complex/ (3771)

13 Lewy Bodies/ (1827)

14 Lewy Bod*.mp. (10361)

15 Cognition Disorders/ (64368)

16 Cognition Disorders/di [Diagnosis] (21205)

17 (Cognition disorder* or disorder* cognition).kf,tw. (466)

18 Memory Disorders/ (21245)

19 (Memory disorder* or age related memory disorder* or memory deficit* or memory disorder*, age related).kf,tw. (13027)

20 Neurodegenerative Diseases/ (18094)

21 (Neurodegenerative disease* or degenerative condition*, neurologic or degenerative neurological disease* or degenerative neurological disorder* or neurologic degenerative condition*).mp. (59029)

22 Alzheimer Disease/ (94779)

23 ((Alzheimer adj2 (disease* or syndrome or dementia*)) or Alzheimer disease, early onset or Alzheimer disease, late onset or Alzheimer type dementia or Alzheimer type senile dementia or Alzheimer's disease* or dementia, Alzheimer or early onset Alzheimer disease* or late onset Alzheimer disease*).mp. (157267)

24 Neurocognitive Disorders/ (9234)

25 (Neurocognitive disorder* or neurocognitive disease*).mp. (11721)

26 or/8-25 (356876)

27 meta-analysis.pt. (120242)

28 meta-analysis/ or systematic review/ or meta-analysis as topic/ or "meta analysis (topic)"/ or "systematic review (topic)"/ or exp technology assessment, biomedical/ (226258)

29 ((systematic* adj3 (review* or overview*)) or (methodologic* adj3 (review* or overview*))).ti,ab,kf,kw. (202807)

30 ((quantitative adj3 (review* or overview* or synthes*)) or (research adj3 (integrati* or overview*))).ti,ab,kf,kw. (11422)

31 ((integrative adj3 (review* or overview*)) or (collaborative adj3 (review* or overview*)) or (pool* adj3 analy*)).ti,ab,kf,kw. (27954)

32 (data synthes* or data extraction* or data abstraction*).ti,ab,kf,kw. (28269)

33 (handsearch* or hand search*).ti,ab,kf,kw. (9546)

34 (mantel haenszel or peto or der simonian or dersimonian or fixed effect* or latin square*).ti,ab,kf,kw. (27311)

35 (met analy* or metanaly* or technology assessment* or HTA or HTAs or technology overview* or technology appraisal*).ti,ab,kf,kw. (9738)

36 (meta regression* or metaregression*).ti,ab,kf,kw. (9340)

37 (meta-analy* or metaanaly* or systematic review* or biomedical technology assessment* or bio-medical technology assessment*).mp,hw. (322260)

38 (medline or cochrane or pubmed or medlars or embase or cinahl).ti,ab,hw. (233774)

39 (cochrane or (health adj2 technology assessment) or evidence report).jw. (19813)

40 (meta-analysis or systematic review).mp. (300106)

41 (comparative adj3 (efficacy or effectiveness)).ti,ab,kf,kw. (14003)

42 (outcomes research or relative effectiveness).ti,ab,kf,kw. (9631)

43 ((indirect or indirect treatment or mixed-treatment) adj comparison*).ti,ab,kf,kw. (2259)

44 or/27-43 (491555)

45 7 and 26 and 44 (116)

46 limit 45 to english language (110)

****

Database: APA PsycInfo <1806 to September Week 4 2020>

Search Strategy:

----

1 primary health care/ (18474)

2 general practitioners/ or physicians/ or family medicine/ or family physicians/ (29513)

3 ((primary care or primary health care or primary healthcare or primary or general practice or general) adj2 (doctor* or physician* or practitioner* or practice*)).ti,ab,hw. (23845)

4 (((family or general) adj2 (practice* or medicine or doctor* or physician* or practitioner* or practice physician)) or physicians, family or general medicine or general medical practice*).ti,ab,hw. (27591)

5 or/1-4 (63299)

6 dementia/ or neurocognitive disorders/ or aids dementia complex/ or dementia with lewy bodies/ or alzheimer's disease/ or cognitive impairment/ (100907)

7 (dementia* or Lewy bod*).ti,ab,hw. (67476)

8 (Cognition disorder* or disorder*, cognition or cognitive disorder* or cognitive defect* or cognitive disability or cognitive dysfunction or cognitive impairment).ti,ab,hw. (55960)

9 (Neurocognitive disorder* or neurocognitive disease*).ti,ab,hw. (1322)

10 ((Alzheimer adj2 (disease* or syndrome or dementia*)) or Alzheimer disease, early onset or Alzheimer disease, late onset or Alzheimer type dementia or Alzheimer type senile dementia or Alzheimer's disease* or dementia, Alzheimer or early onset Alzheimer disease* or late onset Alzheimer disease* or dementia, Alzheimer).ti,ab,hw. (59774)

11 vascular dementia/ (2131)

12 memory disorders/ (4399)

13 (Memory disorder* or age related memory disorder* or memory deficit* or memory disorder*, age related or memory defect or memory impairment*).ti,ab,hw. (17622)

14 neurodegenerative diseases/ (6112)

15 (Neurodegenerative disease* or degenerative condition*, neurologic or degenerative neurological disease* or degenerative neurological disorder* or neurologic degenerative condition*).ti,ab,hw. (12461)

16 or/6-15 (152987)

17 meta-analysis.ti,ab,hw. (30479)

18 meta-analysis/ or systematic review/ or meta-analysis as topic/ or "meta analysis (topic)"/ or "systematic review (topic)"/ or exp technology assessment, biomedical/ (5189)

19 ((systematic* adj3 (review* or overview*)) or (methodologic* adj3 (review* or overview*))).ti,ab,hw. (37282)

20 ((quantitative adj3 (review* or overview* or synthes*)) or (research adj3 (integrati* or overview*))).ti,ab,hw. (9435)

21 ((integrative adj3 (review* or overview*)) or (collaborative adj3 (review* or overview*)) or (pool* adj3 analy*)).ti,ab,hw. (4958)

22 (data synthes* or data extraction* or data abstraction*).ti,ab,hw. (2617)

23 (handsearch* or hand search*).ti,ab,hw. (1286)

24 (mantel haenszel or peto or der simonian or dersimonian or fixed effect* or latin square*).ti,ab,hw. (4847)

25 (met analy* or metanaly* or technology assessment* or HTA or HTAs or technology overview* or technology appraisal*).ti,ab,hw. (867)

26 (meta regression* or metaregression*).ti,ab,hw. (1764)

27 (meta-analy* or metaanaly* or systematic review* or biomedical technology assessment* or bio-medical technology assessment*).mp,hw. (61616)

28 (medline or cochrane or pubmed or medlars or embase or cinahl).ti,ab,hw. (25518)

29 (cochrane or (health adj2 technology assessment) or evidence report).jw. (0)

30 (meta-analysis or systematic review).md. (45469)

31 (comparative adj3 (efficacy or effectiveness)).ti,ab,hw. (2031)

32 (outcomes research or relative effectiveness).ti,ab,hw. (3558)

33 ((indirect or indirect treatment or mixed-treatment) adj comparison*).ti,ab,hw. (200)

34 or/17-33 (102059)

35 5 and 16 and 34 (86)

36 limit 35 to english language (83)

****

Database: Embase <1974 to 2020 October 01>

Search Strategy:

----

1 primary health care/ (66908)

2 general practitioner/ (99815)

3 ((primary care or primary health care or primary healthcare or primary or general practice or general) adj2 (doctor* or physician* or practitioner*)).kw,tw. (106721)

4 general practice/ (78200)

5 (((family or general) adj2 (practice* or medicine or doctor* or physician* or practitioner* or practice physician*)) or physicians, family or general medicine or general medical practice).kw,tw. (168423)

6 or/1-5 (308500)

7 dementia/ or alzheimer disease/ or diffuse lewy body disease/ or hiv associated dementia/ (295351)

8 (dementia* or Lewy bod* or (Alzheimer adj2 (disease* or syndrome or dementia*)) or Alzheimer disease, early onset or Alzheimer disease, late onset or Alzheimer type dementia or Alzheimer type senile dementia or Alzheimer's disease* or dementia, Alzheimer or early onset Alzheimer disease* or late onset Alzheimer disease* or dementia, Alzheimer).kw,tw. (308586)

9 multiinfarct dementia/ (12376)

10 cognitive defect/ (167492)

11 (Cognition disorder* or disorder*, cognition or (cognitive adj2 (disorder* or defect* or disability or dysfunction))).kw,tw. (37759)

12 memory disorder/ (36378)

13 (Memory disorder* or age related memory disorder* or memory deficit* or memory disorder*, age related or memory defect or memory impairment*).kw,tw. (33208)

14 degenerative disease/ (55246)

15 (Neurodegenerative disease* or degenerative condition*, neurologic or degenerative neurological disease* or degenerative neurological disorder* or neurologic degenerative condition*).kw,tw. (65950)

16 "disorders of higher cerebral function"/ (1575)

17 (Neurocognitive disorder* or neurocognitive disease* or disorders of higher cerebral function).kw,tw. (4533)

18 or/7-17 (584290)

19 meta analysis.kw,tw. (208242)

20 meta-analysis/ or systematic review/ or meta-analysis as topic/ or "meta analysis (topic)"/ or "systematic review (topic)"/ or exp technology assessment, biomedical/ (422807)

21 ((systematic* adj3 (review* or overview*)) or (methodologic* adj3 (review* or overview*))).ti,ab,kw. (252387)

22 ((quantitative adj3 (review* or overview* or synthes*)) or (research adj3 (integrati* or overview*))).ti,ab,kw. (13414)

23 ((integrative adj3 (review* or overview*)) or (collaborative adj3 (review* or overview*)) or (pool* adj3 analy*)).ti,ab,kw. (39787)

24 (data synthes* or data extraction* or data abstraction*).ti,ab,kw. (34871)

25 (handsearch* or hand search*).ti,ab,kw. (11418)

26 (mantel haenszel or peto or der simonian or dersimonian or fixed effect* or latin square*).ti,ab,kw. (35678)

27 (met analy* or metanaly* or technology assessment* or HTA or HTAs or technology overview* or technology appraisal*).ti,ab,kw. (15520)

28 (meta regression* or metaregression*).ti,ab,kw. (11730)

29 (meta-analy* or metaanaly* or systematic review* or biomedical technology assessment* or bio-medical technology assessment*).mp,hw. (510796)

30 (medline or cochrane or pubmed or medlars or embase or cinahl).ti,ab,hw. (305608)

31 (cochrane or (health adj2 technology assessment) or evidence report).jw. (27150)

32 (meta-analysis or systematic review).mp. (478480)

33 (comparative adj3 (efficacy or effectiveness)).ti,ab,kw. (20159)

34 (outcomes research or relative effectiveness).ti,ab,kw. (13765)

35 ((indirect or indirect treatment or mixed-treatment) adj comparison*).ti,ab,kw. (4192)

36 or/19-35 (711330)

37 6 and 18 and 36 (343)

38 limit 37 to english language (326)

****

Database: EBM Reviews - Cochrane Database of Systematic Reviews <2005 to October 1, 2020>

Search Strategy:

----

1 Primary Health Care.mp. [mp=title, abstract, full text, keywords, caption text] (197)

2 Physicians, primary care.mp. [mp=title, abstract, full text, keywords, caption text] (16)

3 General practitioner.mp. [mp=title, abstract, full text, keywords, caption text] (374)

4 ((primary care or primary health care or primary healthcare or primary or general practice or general) adj2 (doctor* or physician* or practitioner* or practice*)).ab,kw,ti. (76)

5 Family practice.mp. [mp=title, abstract, full text, keywords, caption text] (99)

6 General practice.mp. [mp=title, abstract, full text, keywords, caption text] (422)

7 (((family or general) adj2 (practice* or medicine or doctor* or physician* or practitioner* or practice physician)) or physicians, family or general medicine or general medical practice*).ab,kw,ti. (65)

8 or/1-7 (840)

9 Dementia.mp. [mp=title, abstract, full text, keywords, caption text] (541)

10 Dementia, Multi-Infarct.mp. [mp=title, abstract, full text, keywords, caption text] (2)

11 AIDS Dementia Complex.mp. [mp=title, abstract, full text, keywords, caption text] (3)

12 Lewy Bodies.mp. [mp=title, abstract, full text, keywords, caption text] (61)

13 dementia*.ab,kw,ti. (234)

14 Lewy bod*.ab,kw,ti. (9)

15 Cognition disorders.mp. [mp=title, abstract, full text, keywords, caption text] (89)

16 (Cognition disorder* or disorder*, cognition or (cognitive adj2 (disorder* or defect* or disability or dysfunction or impairment))).ab,kw,ti. (223)

17 Memory Disorders.mp. [mp=title, abstract, full text, keywords, caption text] (26)

18 (Memory disorder* or age related memory disorder* or memory deficit* or memory disorder*, age related or memory defect or memory impairment*).ab,kw,ti. (15)

19 Neurodegenerative Diseases.mp. [mp=title, abstract, full text, keywords, caption text] (41)

20 (Neurodegenerative disease* or degenerative condition*, neurologic or degenerative neurological disease* or degenerative neurological disorder* or neurologic degenerative condition*).ab,kw,ti. (7)

21 Alzheimer Disease.mp. [mp=title, abstract, full text, keywords, caption text] (85)

22 ((Alzheimer adj2 (disease* or syndrome or dementia*)) or Alzheimer disease, early onset or Alzheimer disease, late onset or Alzheimer type dementia or Alzheimer type senile dementia or Alzheimer's disease* or dementia, Alzheimer or early onset Alzheimer disease* or late onset Alzheimer disease* or dementia, Alzheimer).ab,kw,ti. (95)

23 Neurocognitive Disorders.mp. [mp=title, abstract, full text, keywords, caption text] (14)

24 (Neurocognitive disorder* or neurocognitive disease*).ab,kw,ti. (3)

25 or/9-24 (612)

26 8 and 25 (83)

****
